# A review on potential neurotoxicity of titanium dioxide nanoparticles

**DOI:** 10.1186/s11671-015-1042-9

**Published:** 2015-08-26

**Authors:** Bin Song, Jia Liu, Xiaoli Feng, Limin Wei, Longquan Shao

**Affiliations:** Guizhou Provincial People’s Hospital, Guiyang, 550002 China; Nanfang Hospital, Southern Medical University, Guangzhou, 510515 China

**Keywords:** Titanium dioxide, TiO_2_, Anatase, Rutile, Nanoparticles, Brain, CNS, Neurotoxicity

## Abstract

As the rapid development of nanotechnology in the past three decades, titanium dioxide nanoparticles (TiO_2_ NPs), for their peculiar physicochemical properties, are widely applied in consumer products, food additives, cosmetics, drug carriers, and so on. However, little is known about their potential exposure and neurotoxic effects. Once NPs are unintentionally exposed to human beings, they could be absorbed, and then accumulated in the brain regions by passing through the blood–brain barrier (BBB) or through the nose-to-brain pathway, potentially leading to dysfunctions of central nerve system (CNS). Besides, NPs may affect the brain development of embryo by crossing the placental barrier. A few in vivo and in vitro researches have demonstrated that the morphology and function of neuronal or glial cells could be impaired by TiO_2_ NPs which might induce cell necrosis. Cellular components, such as mitochondrial, lysosome, and cytoskeleton, could also be influenced as well. The recognition ability, spatial memory, and learning ability of TiO_2_ NPs-treated rodents were significantly impaired, which meant that accumulation of TiO_2_ NPs in the brain could lead to neurodegeneration. However, conclusions obtained from those studies were not consistent with each other as researchers may choose different experimental parameters, including administration ways, dosage, size, and crystal structure of TiO_2_ NPs. Therefore, in order to fully understand the potential risks of TiO_2_ NPs to brain health, figure out research areas where further studies are required, and improve its bio-safety for applications in the near future, how TiO_2_ NPs interact with the brain is investigated in this review by summarizing the current researches on neurotoxicity induced by TiO_2_ NPs.

## Review

### Introduction

Nanomaterial, with one dimension in the range of 1 to 100 nm at least, possesses unique physicochemical [1], optical [2], and electrical properties [3]. Because of its peculiar features, nanomaterial is widely applied in cosmetics [4], food and personal care products [5], medical devices [6], and so on. As nanotechnology is advancing rapidly, more concerns on health risks about exposure to nanoparticles have been arising. The TiO_2_ particles are believed to possess low toxicity and thus are widely used in biomedical applications for their excellent biocompatibility [7–9]. However, when the size of TiO_2_ is diminished to nanoscale, the bioactivity and physiochemical properties of nano-sized TiO_2_ are significantly different from the properties of their bulk analogue. As a consequence, the toxic effects of TiO_2_ NPs on human beings could not be simply determined by traditional methods. What’s more, the understanding about the risk assessments of NPs is insufficient and often lags behind their rapid advancement and widespread applications [10–13]. TiO_2_ NPs-containing products are widely used as well, which could unintentionally lead to human exposure and environmental pollution. The TiO_2_ NPs might be potentially absorbed mainly through inhalation, indigestion, and skin penetrations into the circulation of human beings. And then they may be redistributed into other tissues (such as the liver, heart, lung, etc.), which could induce impairments on organs after unintentional exposure. As the concerns about unintentional exposure of NPs on human beings arise, an increasing number of researches have been performed to study the potential toxic effects of TiO_2_ NPs in recent years. Several in vivo researches adopted rats or mice for the experimental models, and they were exposed to TiO2 NPs for bio-safety assessment. The main administration routes in in vivo studies included inhalation [14], intratracheal [15] or nasal instillation [16], oral gavage and dermal exposure [17], intragastric feeding [18], intraperitoneal [19], and intravenous injection [15]. Numerous reports have revealed that when the TiO_2_ NPs were administrated and transported into second targets, they could induce renal fibrosis, change cell cycle of lung epithelial cell, disturb the metabolism of hepatocytes, and impair the spleen [20–23].

The central nervous system (CNS), including the brain and spinal cord, is an extremely important system for human beings. Its functions are mainly composed of the following: (1) to transfer, store, and process information; (2) to generate a variety of psychological activities; and (3) to command and control all the behaviors of human beings. Several in vivo studies have investigated the bio-distribution of TiO_2_ NPs. After rats or mice were exposed to TiO_2_ NPs, the NPs were capable of reaching most parts of the brain zones, and the Ti contents in the brain were increased. The main pathways for TiO_2_ NPs to be transported into the brain included (1) the blood–brain barrier (BBB) pathway [24], (2) the olfactory nerve translocation pathway [25], and (3) the placental barrier pathway [26]. However, the processes of translocation of TiO_2_ NPs into the brain would be regulated by several parameters, such as administration routes, size, and surface modification. Once the TiO_2_ NPs were transported into the brain regions, major cells in the CNS, including the neurons and the glial cells, would be affected by NPs. Reactive oxygen species (ROS), apoptosis, and inflammation would be induced by TiO_2_ NPs, which may lead to cell death and disturb the CNS functions or even induce neurodegenerative disease. Some in vitro studies also indicated that when neurons or glial cells were incubated with TiO_2_ NPs, the viability, cell cycle, cell morphology, antioxidant capability, and cellular components would be affected [27–30].

However, current knowledge about neurotoxicity induced by TiO_2_ NPs is insufficient and more detailed and standardized researches are needed. Therefore, in order to fully understand the potential risks of TiO_2_ NPs to brain health, figure out research areas where further studies are required, and improve its bio-safety for applications in the near future, how TiO_2_ NPs can be translocated into the brain and how they influenced the CNS function are investigated in this review via summarizing relevant in vivo and in vitro researches.

### The main routes of TiO_2_ NPs into the brain

Due to peculiar physicochemical properties of NPs [31], TiO_2_ NPs are widely used in many fields, such as photovoltaic appliances [32], sensors [33], renewable energy devices [34], functional building blocks [35], textiles [10], sunscreens [11], food [13], and medical applications [36]. These widespread applications, however, put humans at a high risk of getting exposed to TiO_2_ NPs, probably through inhalation, ingestion, skin penetration, medical applications, and so on. Therefore, it is urgent to evaluate the bio-safety of TiO_2_ NPs at length. The CNS is an extremely important system for human beings. Numerous studies have already demonstrated that once NPs were absorbed, they were able to be transported to the second targets, including the brain, liver, lung, spleen, and so on. In in vivo studies, after the experimental rodents were exposed to TiO_2_ NPs, these NPs can be transported into the brain regions mainly through the following routes.

#### Translocation of TiO_2_ NPs from the blood to the brain

**Fig. 1:**
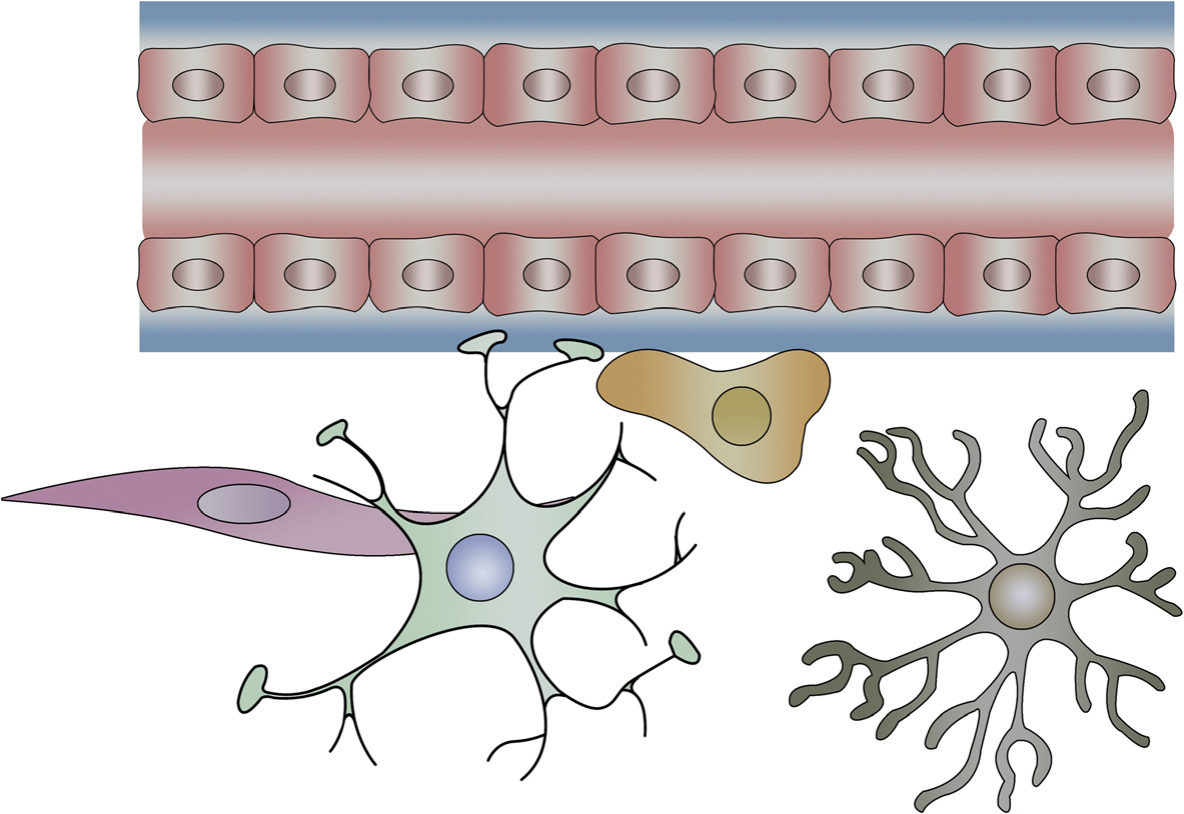
A diagram of the blood–brain barrier structure

The BBB is an effectively protective structure, which is mainly composed of endothelial cells, astrocytes, and pericytes [37]. The endothelial cells are connected with each other through complicated tight junctions, while the connections are supported by the astrocytes and pericytes. On account of this sophisticated structure, only specific substances with small size or low-molecular weight (MW) could be allowed to pass through the BBB by means of three main transport patterns (passive diffusion, active transport, and endocytosis). In another word, BBB is capable of protecting the healthy and functional CNS from being affected by harmful chemicals, toxins, and drugs in the circulatory system. Whereas, NPs possess unique chemical–physical characteristics and tiny size which make them be similar to biomolecule. Therefore, they are able to pass through the BBB and enter into the CNS [38–40] (Fig. 1). On the other hand, the permeability of BBB can be altered by NPs, which could assist in influx of exogenous substances into the brain. As a result, NPs induced inflammation, edema, and cell injury or even cell death in brain regions.

In this in vivo study [41], TiO_2_ NPs with 3 nm diameter were repeatedly administrated on mice through intratracheal instillations in a chronic way. After 4 weeks, compared with the control group, the percentage of brain-to-body weight was downregulated. Through histopathological examination, inflammatory cell aggregation and cell necrosis were presented in the brain zones. The amount of Ti in the brain was upregulated measured by inductively coupled plasma mass spectrometry (ICP-MS). These results indicated that (1) after mice were intratracheally instilled with TiO_2_ NPs, those NPs were transported into the blood, then they passed through the BBB, and finally accumulated in the brain. (2) TiO_2_ NPs which were transported into the CNS could induce its injury. In another research [42], rats were treated with TiO_2_ NPs suspension of different diameters (10, 20, and 200 nm) through aerosol inhalation. Seventy two hours later, TiO_2_ NPs with diameters of 10 and 20 nm were both transported into the brain, inducing cerebral injury in a dosage-dependent way. However, TiO_2_ NPs with diameter of 200 nm did not cause any significant changes in the brain. From these results, we could infer that the ability of passing through the BBB for TiO_2_ NPs was associated with the nanoparticle diameter.

TiO_2_ NPs could not only pass through the BBB but also disrupt the integrity of the BBB. The toxic effects of early, acute, and long-term exposures of TiO_2_ NPs on the BBB were investigated on the basis of an in vitro BBB model. The model, mimicking the specific characteristics of an in vivo one [43], was composed of rat primary endothelial cells (BECs) and astrocytes. After the BBB model was treated with TiO_2_ NPs for acute or long-term exposure, the expression levels of P-glycoprotein (P-gp), claudin 5, caveolin-1, and caveolin-2, which regulated the integrity of BBB, were reduced. These results indicated that direct harmful impacts of TiO_2_ NPs on BBB integrity were presented during the acute and long-term exposure. While in this study, authors still discovered that after exposure for 4 h the mRNA expression levels of CXC chemokines, CC chemokines, ADAM17, Ccl2, Tgfβ1, ICAM, and VCAM were increased, paralleled by the decreased mRNA expression levels of ABC transporters. The upregulated expression levels of those target genes were reported to be related with the decreased permeability of BBB [44–50], which could facilitate the transportation of other exogenous substances into brain. These findings meant that besides direct impairments, the indirect harmful impacts (inflammatory effects) of TiO_2_ NPs on BBB integrity also occurred. On the other hand, if the NPs were eliminated from the brain slowly, they might induce long-term adverse effects after the exposure.

#### Axonal translocation of TiO_2_ NPs from the nose to the brain

**Fig. 2:**
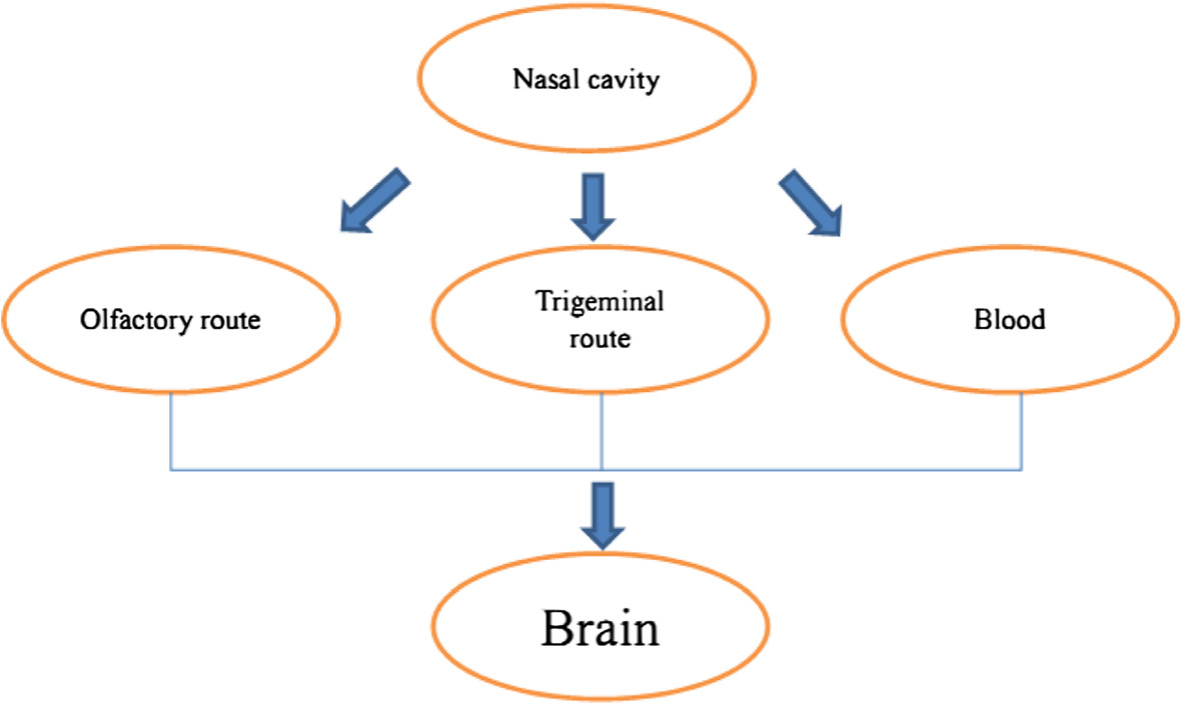
A simple diagram of nose–brain pathway after intranasal administration

Axonal transport is defined as the process that proteins and other substances synthesized in neurosome which are transported to the nerve endings through the cytoskeleton [51]. However, some low-molecular weight or small-size substances such as NPs could be taken up by the nerve ending, and then transported to the neurosome. This process is called retrograde axonal transport [52]. The olfactory and trigeminal nerve endings are abundant in the nasal areas. Once NPs were instilled through the nose, they can enter the circulation system and pass through the BBB, or they can bypass the BBB to be transported into the brain regions along the axons. The second direct pathway is reported to be the major route for NPs of being transported to the brain zones after intranasal instillation (Fig. 2).

Wang et al. [16] confirmed that after the female mice were exposed to TiO_2_ NPs of different sizes with two crystal types (80 nm for rutile and 155 nm for anatase) through intranasal instillation, the Ti concentration was significantly increased in the brain as compared with the control. Wang et al. [25] discovered that when female mice were treated with TiO_2_ NPs (80 nm rutile, 155 nm anatase) through intranasal instillation every other day, the titanium content was detected in the brains after 30 days. The Ti contents determined by synchrotron radiation X-ray fluorescence (SRXRF) analysis and ICP–MS were significantly upregulated in the hippocampus, followed by the olfactory bulb, cerebellum, and cerebral cortex. These findings suggested that the TiO_2_ NPs could be transported to the mice brain through the olfactory bulb after intranasal instillation. The same research group conducted another study [53], which also detected Ti contents in the hippocampus, olfactory bulb, cerebellum, and cerebral cortex by ICP-MS in a time-dependent way. Both the two studies indicated that there was a high Ti content in the hippocampus, so this brain area would be easily affected by TiO_2_ NPs. It is generally accepted that the hippocampus is mainly in charge of memory and learning [54, 55]. Therefore, impairments on it might probably induce neurodegenerative diseases, such as Alzheimer’s diseases [56–58].

In another in vivo study [59], the female mice were treated with TiO_2_ NPs by intranasal instillation. The experimental mice were divided into four groups according to different sizes, coatings, and shapes of the TiO_2_ particles. Groups A and B shared the same insoluble property, but with various size (micro- and nano-sized TiO_2_ particles) and no surface coatings. Groups C and D were hydrophilic NPs and silica-coated with different shapes. After treatment every other day for 30 days, the titanium contents in the brain were determined by ICP-MS. These data demonstrated that groups C and D showed higher Ti concentration in the cerebral cortex and striatum than groups A and B, while group A was detected with no Ti content in the sub-brain zones. These results indicated that the size, surface modification, and shape of TiO_2_ NPs played an important role on their transport to the brain from the nose. In this study [60], CD-1 (ICR) female mice were exposed to TiO_2_ NPs with different dosages (2.5, 5, and 10 mg/kg body weight). After nasal administration for 90 consecutive days, the TiO_2_ NPs were detected in the brain, and this accumulation could induce CNS dysfunctions.

#### Translocation into the brain of offspring through the placental barrier

**Fig. 3:**
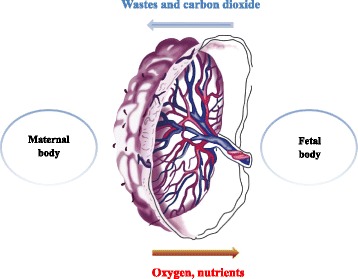
Substance exchange between the mother and fetus through placenta barrier

Placental barrier, composed of both maternal and fetal tissues, is another internal barrier that can protect the development of embryo [61]. It could protect the fetus from being affected by harmful substances in maternal blood circulation, while the fetus could get nutrients and oxygen from the mother via the placenta (Fig. 3). However, a great number of studies [62, 63] have already revealed that after pregnant mice/rats were exposed to exogenous substances, such as nanoparticles, those substances could be detected in the brain of fetus, and then they can disturb the homeostasis of CNS or even induce neuronal death. Those harmful impacts on fetus brain have been demonstrated to be related with psychiatric disorders such as autism, schizophrenia, depression, and so on in their later life [64, 65]. As a consequence, those findings suggest that placental barrier plays an important part on fetal growth and development.

In this study [66], pregnant Wistar rats were administrated with TiO_2_ NPs intragastrically daily from gestational day 2 to 21. Then, the 1-day-old neonates were sacrificed, and the TiO_2_ NPs concentration in the hippocampus, determined by ICP-MS, was significantly upregulated as compared with the control group. Yamashita et al. [67] also detected silica (70 nm) and TiO_2_ (35 nm) NPs in the placenta and fetal brain after the pregnant mice were injected intravenously with these NPs, which led to the pregnancy complication.

In another study [68], the authors discovered that when pregnant ICR mice were treated with TiO_2_ NPs, the levels of dopamine and its metabolites were increased in some regions of the fetus brain on postnatal day 21 as compared with the control group. Another study [69] adopted microarray to assess gene expression changes in the brains of male fetus and pups after the pregnant ICR mice were exposed to TiO_2_ NPs. Data showed that the expression levels of genes related with dopamine neuron system were altered. Microarray was also applied in this study [70], and pregnant ICR mice were administrated with anatase TiO_2_ NPs. By analyzing the gene expression alternations in the brains of male fetus and pups, data obtained revealed that the expression levels of genes related with oxidative stress, neurotransmitters, and psychiatric diseases were dysregulated. The neurobehavioral performance of the offspring might be moderately altered due to maternal exposure to TiO_2_ NPs [71]. Similarly, Cui et al. [72] discovered that when Sprague–Dawley rats were injected subcutaneously with TiO_2_ NPs, the antioxidant ability of pups’ brain was impaired. Although these researches did not measure the contents of TiO_2_ NPs in the brain directly, those data collected indirectly demonstrated that the TiO_2_ NPs in maternal circulation system would affect the development of embryo. Then, they could impair the brain development and finally lead to CNS dysfunctions in their later life.

### Bio-distribution and elimination of TiO_2_ NPs from the brain

**Fig. 4:**
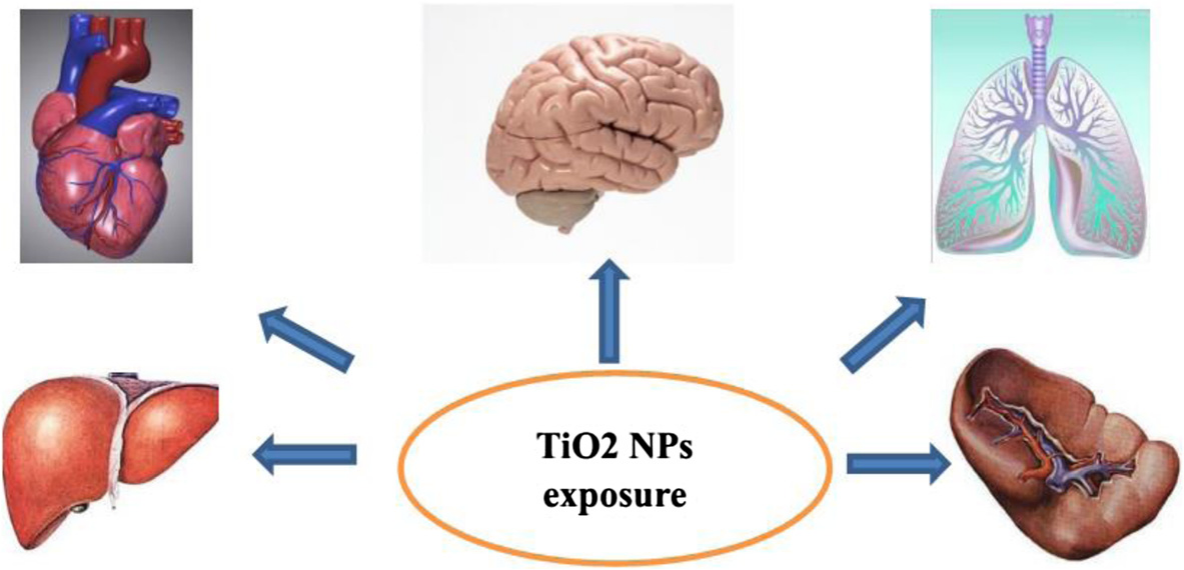
A simple diagram of bio-distribution of Ti after TiO_2_ NPs exposure

When TiO_2_ NPs were absorbed into circulation, they were capable of being redistributed to second organs (Fig. 4). At present, several researches have been performed to study the bio-distribution of TiO_2_ NPs after administrations (Table 1). In this study [73], when rats were treated with TiO_2_ NPs (5 mg/kg body weight) by intravenous injection, TiO_2_ NPs can be detected in the liver, spleen, lung, and kidney except blood cells, plasma, brain, and lymph nodes. The BALB/c female mice were exposed to TiO_2_ NPs at a dose of 560 mg/kg by intravenous injection (i.v.) or 5600 mg/kg by subcutaneous injection (s.c.). The TiO_2_ NPs were detected by energy dispersive X-ray spectroscopy (EDS) in the lung, liver, lymph node, spleen, and kidney from i.v.-administrated mice but only in the liver, lymph node, and spleen of s.c.-administrated mice, while the content of NPs was not detected in the brain [74]. Another study [75] also did not detect TiO_2_ NPs in the brain of male mice except blood and liver after i.v. injection. However, after hairless mice were treated with TiO_2_ NPs (21 nm) by dermal exposure for 60 days, significant pathological alterations were presented in the skin and liver and the NPs were also detected in the brain without pathological changes [76]. Wang et al. [16] studied the bio-distribution of TiO_2_ NPs (50 mg/kg) after female mice were treated with NPs by intranasal instillation every other day for 30 days. The biochemical parameters of the liver, spleen, heart, and serum were not affected by NPs as compared with the control group; while the concentration of NPs was apparently enhanced in the lung and brain regions. Another study investigated the bio-distribution of TiO_2_ NPs after rats were repeatedly orally administrated for 13 weeks. Even in the highest dosage group (1041.5 mg/kg BW), the Ti content in the brain was minimal with no statistical significance. Geraets et al. [77] compared the different distributions of TiO_2_ NPs in rats after oral and intravenous administration. The data obtained demonstrated that the Ti concentrations were not detectable in tissues, including the brain after oral administration. However, the Ti contents were detected in the liver, spleen, kidney, lung, heart, brain, thymus, and reproductive organs after intravenous injection. It could be inferred from those studies that (1) intranasal instillation might be the most effective routes for TiO_2_ NPs transported to the brain and (2) Ti content could be undetectable in the brain regions after intravenous injection. Undoubtedly, those conclusions drawn from abovementioned in vivo researches might not be convincing, because the translocations of TiO_2_ NPs into the brain would be influenced by several parameters, such as administration routes, size, dosage, and so on, which would be discussed in later chapters.

**Table 1:** Bio-distribution of TiO_2_ NPs after rat/mice were administrated by different routes

Crystal type	Animal	Administration	Parameters/dose	Bio-distribution	Reference
Both anatase and rutile forms (70/30)	Male Wistar rats	Intravenous injection	20–30 nm; no surface coating; 5 mg/kg body weight (BW); single injection	Liver, spleen, lung, and kidney detected; blood cells, plasma, lymph nodes, and brain not detected	[73]
Both anatase and rutile forms (80/20)	BALB/c female mice	Subcutaneous (s.c.) injection	Hydrodynamic diameters ranging from 114 to 122 nm; 5600 mg/kg BW; 2 consecutive days	Liver, lymph node and spleen detected; brain not detected	[74]
		Intravenous (i.v.) injection	Hydrodynamic diameters ranging from 114 to 122 nm; 560 mg/kg BW; 2 consecutive days	Lung, liver, lymph node, spleen and kidney detected; brain not detected	
Rutile	Male mice	Intravenous injection	Primary particle diameter 15 nm, secondary particle size 120 nm; 1813 μg/animal	Liver, kidney, blood detected; brain not detected	[75]
Rutile	CD-1 (ICR) female mice	Intranasal instillation	80 nm; 50 mg/kg BW; every other day for 30 days	Lung and brain detected; liver, heart, and spleen not detected	[16]
Anatase			155 nm; 50 mg/kg BW; every other day for 30 days		
Degussa P25	Rats	Intravenous administration	21 nm; spherical; 0.95 mg/kg BW; single injection	Liver (highest), spleen, lung, kidney, heart and blood detected; brain not detected	[113]
Anatase 80: 20 rutile	Sprague–Dawley rats	Oral administration	21 nm; spherical; 260.4, 520.8, and 1041.5 mg/kg/day BW; every day for 13 weeks (7 days/week)	Low absorption in other organs and brain not detected	[78]
Anatase	Male Kunming mice	Inhalation exposure	20 nm; steady concentration (6.34 ± 0.22 mg m^−3^); 8 h per day for 3 weeks	Lungs, liver, blood, and urine detected; kidney and brain not detected	[114]

Although TiO_2_ NPs are capable of entering into the brain regions through specific routes by a variety of delivery ways, the capability of excretion would keep the brain from being affected by NPs. But few researches about the elimination of TiO_2_ NPs from the brain were published. In Cho et al.’s study [78], when the Sprague–Dawley rats were administrated with TiO_2_ NPs by intravenous injection (a single or repeated dosage of 1 ml for 5 consecutive days), the contents of NPs were determined on days 2/6, 14, 30, and 90 after administration. The Ti contents were detected in the liver (the highest), spleen, kidney, lung, heart, brain, thymus, and reproductive organs on day 2/6. During the observation period, Ti concentrations in the feces and urine demonstrated no increase as compared with control group. Also, Ti contents in the brain demonstrated no detectable alterations.

It can be inferred from the results that (1) the excretion of TiO_2_ NPs from the brain is limited and (2) even negligible dosage of TiO_2_ NPs could be accumulated in the brain with minimal elimination, so chronic or long-term exposure might be potentially risky for the brain health. TiO_2_ NPs are thought to be low toxic. However, the long-term, repeated exposure, and durative existence of TiO_2_ NPs in the brain regions made it necessary to re-evaluate their bio-safety. Therefore, more in vivo researches are needed to further investigate the parameters of TiO_2_ NPs which might influence their elimination rates from the brain. Then, the health risks of TiO_2_ NPs to the brain are expected to be lowered.

### Neurotoxicity of titanium dioxide nanoparticle

Once the TiO_2_ NPs are translocated into the CNS through the abovementioned pathways, they may accumulate in the brain regions. For their slow elimination rates, those NPs could remain in the brain zones for a long period, and the Ti contents would gradually increase with repeated exposure. This would induce pathologic changes, such as inflammation, immunological response, edema, cell injury, cell necrosis, and so on, which would ultimately lead to CNS dysfunctions, including neurodegenerative diseases and psychiatric disorders. Generally, the neurons and glial cells are the main cell types in the CNS. Therefore, the toxic effects of TiO_2_ NPs on them would lead to impairments on the brain as a consequence.

#### Toxic effects on CNS in in vivo studies

Several in vivo studies have demonstrated that the TiO_2_ NPs could be transported into the brain regions, and then accumulated in the CNS, eventually leading to CNS dysfunctions (Table 2).

**Table 2:** Toxic effects of TiO2 NPs on CNS in in vivo studies

Crystal type	Animals	Cell type	Parameters/dose	Main findings	Reference
Rutile 80 nm anatase 155 nm	CD-1 (ICR) female mice	Nasal instillation	500 μg; every other day for 30 days	Ti contents detected in the brain; GFAP-positive cell, CAT, SOD, MDA, protein carbonyls, AChE activities, glutamic acid, and NO increased	[25]
Anatase bulk	CD-1 (ICR) female mice	Delivered to the abdominal cavity	5 nm; 5, 10, 50, 100, 150 mg/kg BW; every day for 14 days	Ti contents detected in brain; O_2_, H_2_O_2_, MDA, NOS, NO increased; Glu contents, antioxidative enzymes, non-enzymatic antioxidant contents, and AChE activity decreased	[79]
Anatase	CD-1 male mice	Intranasal administration	5–6 nm; 2. 5, 5, 10 mg/kg BW. every day for 90 days	Ti contents detected in brain; no daily behavioral changes; O_2_, H_2_O_2_, MDA, protein carbonyl, 8-OHdg, p38, JNK, NF-κB, Nrf-2, and HO-1 increased	[80]
Anatase	Sprague–Dawley rats (male and female)	Subcutaneous injection	5 nm; 500 μl (1 μg/μl) on GD 6, 9, 12, 15, and 18	CAT, GSH-PX, and T-AOC decreased; MDA and 8-hydroxydeoxyguanosine (8-OHdG) increased	[72]
Rutile 80 nm anatase 155 nm	CD-1 (ICR) female mice	Intranasal instillation	500 μg; every other day for 30 days; evaluated at 2, 10, 20, and 30 days of post-instillation time points	Ti contents detected in brain; GSH-Px, GST, SOD and GSH not changed; MDA, TNF-α and IL-1β increased	[53]
Anatase	CD-1 (ICR) female mice	Intranasal administration	5–6 nm; 2.5, 5, 10 mg/kg BW; every day for 90 days	TLR2, TLR4, TNF-α, IKK1, IKK2, NF-κB, NF-κBP52, NF-κBP65, NIK, and IL-1β increased; spatial recognition memory and locomotor activity affected	[81]
Rutile	Male C57BL/6 mice	Intraperitoneal injection	Fine (<1 μm), ultrafine (21 nm); 40 mg/kg BW; one injection 30 min after LPS or vehicle injection	IL-1β, TNF-α, iNOS, ROS production, and OX-42 enhanced by ultrafine TiO_2_ in the LPS-stimulated group	[82]
Anatase	CD-1 (ICR) female mice	Intragastric administration	6.5 nm; 5, 10, 50 mg/kg BW; every day for 60 days	Ti contents in the hippocampus increased; caspase-9, caspase-3, Bax, cytochrome c, O_2_ and H_2_O_2_ upregulated; Bcl-2, SOD, CAT, APx, and GSH-Px reduced	[83]
Anatase	CD-1 female mice	Nasal administration	5–6 nm; 2.5, 5, 10 mg/kg BW; every day for 90 days	NR2A, NR2B, CREB-1, CREB-2, FosB/DFosB, CaMKIV, and pCREB decreased	[85]
Anatase	CD-1 female mice	Intragastric administration	5 nm; 5, 10, 50 mg/kg BW; every day for 60 days	Ti contents in brain upregulated; reduction in the activities of Na^+^/K^+^-ATPase, Ca^2+^-ATPase, Ca^2+^/Mg^2+^-ATPase; Ache, Glu, and NO elevated; NE, DA, DOPAC, 5-HT, and 5-HIAA reduced	[86]
Anatase	Pregnant ICR mice	Subcutaneous injection	2570 nm; 100 μg, injection on GD 6, 9, 12, 15	Genes related with cell death, apoptosis, oxidative stress, inflammation and neurotransmitters changed	[70]
Rutile	Pregnant BALB/c mice	Intravenous injection	35 nm; 0.8 mg, injections on GD 16 and 17	Lower uterine weights and smaller fetuses; fetal resorption and retarded fetal growth	[67]
Anatase	Pregnant Wistar rats	Intragastric administration	10 nm; 100 mg/kg BW, every day from GD 2 to 21	Ti contents elevated and Ki-67-positive cells reduced; learning and memory in offspring disrupted	[66]

Wang et al. [25] revealed that after the female mice were treated with TiO_2_ NPs (80 nm, rutile and 155 nm, anatase; 500 μg) through intranasal instillation every other day for 30 days, the Ti contents were detected in the brain regions, including the olfactory bulb, hippocampus, cerebellum, and cerebral cortex. This accumulation induced increased glial fibrillary acidic protein (GFAP)-positive cell, CAT activity, MDA content, protein carbonyls content, AChE activities, glutamic acid, and NO content, accompanied by cell lost in both experimental groups. While, SOD level was increased only in the 155 nm group. In another study [79], the ICR female mice were injected with TiO_2_ NPs (anatase; 5 nm; 5, 10, 50, 100, 150 mg/kg BW) into the abdominal cavity every day for 14 days. The coefficients of the brain-to-body weight, antioxidative enzymes (SOD, CAT, APx, GSH-Px), non-enzymatic antioxidant contents (ASA/DASA,GSH/GSSG), Glu contents, and AChE activity were decreased, but the Ti contents in the brain, the levels of O_2_, H_2_O_2_, MDA, NOS, and NO were increased in a dose-dependent way. Ze et al. [80] discovered that when CD-1 male mice were intranasally treated with TiO_2_ NPs (5–6 nm; 2.5, 5, 10 mg/kg BW) every day for 90 days, the mRNA expressions of genes regulating oxidative stress, including p38, JNK, NF-κB, Nrf-2, and HO-1, were increased besides upregulated Ti concentrations and levels of O_2_, H_2_O_2_, MDA, protein carbonyl, and 8-OHdg,.

However, in another study [53], after the CD-1 (ICR) female mice were exposed to TiO_2_ NPs (rutile 80 nm and anatase 155 nm) every other day for 30 days by intranasal instillation, the activities of GSH-Px, GST, SOD, and GSH level in the brain were not changed at 30 days. Yet, the level of MDA was enhanced all the same. The Ti contents were highest in the hippocampus, followed by olfactory bulb, cerebellum, and cerebral cortex. However, the levels of TNF-α and IL-1β in brain were upregulated in the 155 nm group, which indicated that intranasal instillation with anatase TiO_2_ NPs would induce inflammation in the brain of mice. Ze et al. [81] also demonstrated that TiO_2_ NPs could induce inflammation in mice brain. In their study, the mRNA and protein levels of Toll-like receptor (TLR)2, TLR4, TNF-α, IKK1, IKK2, NF-κB, NF-κBP52, NF-κBP65, NIK, and IL-1β in the brain were enhanced after the female mice were treated with TiO_2_ NPs (5–6 nm; 2.5, 5, 10 mg/kg BW) every day for 90 days. However, the mRNA expression and protein level of IκB were downregulated significantly. Moreover, the spatial recognition memory and locomotor activity were impaired mostly due to the inflammation response to TiO_2_ NPs. In this study [82], ultrafine TiO_2_ (21 nm, 40 mg/kg BW, one injection 30 min after vehicle administration) could not induce detectable impairments on the brain when the male C57BL/6 mice were exposed by intraperitoneal injection. However, when mice were pretreated with lipopolysaccharide (LPS), the mRNA expression levels of IL-1β, TNF-α, and iNOS in the cortex and hippocampus were markedly upregulated at 2 h after LPS injection, accompanied by significantly increased protein level of IL-1β at 6 h after the usage of LPS pretreatment in the LPS-stimulated group. The ROS production and the expression of OX-42 were significantly increased in the cortex and hippocampus by ultrafine TiO_2_ at 24 h after LPS injection in the LPS-treated group. It was inferred from these findings that ultrafine TiO_2_ could augment the damage in the pre-inflamed but not the healthy brain.

Hu et al. [83] revealed that after CD-1 (ICR) female mice were exposed to TiO_2_ NPs (6.5 nm; 5, 10, 50 mg/kg BW) by intragastric administration every day for 60 days, the expressions of apoptosis-related genes were affected. The mRNA and protein expression levels of caspase-9, caspase-3, Bax, and cytochrome c were upregulated with downregulated level of Bcl-2. However, the caspase-8 level was not affected, which indicated that this apoptosis of the hippocampus induced by TiO_2_ NPs might result from intrinsic pathway. Moreover, levels of O_2_ and H_2_O_2_ were increased and the activities of SOD, CAT, APx, and GSH-Px were downregulated. The ratios of AsA to DAsA and GSH to GSSGG were decreased as well. These results indicated that the antioxidant capabilities of the brain were impaired by TiO_2_ NPs. The time spending exploring the novel arm was significantly reduced in the experimental groups as compared with the control one, which inferred that the spatial recognition memory of mice was impaired for the apoptosis of neurons in hippocampus. In this study [84], when Wistar rats were injected intravenously with nano-TiO_2_ (21 nm; 5, 25, and 50 mg/kg BW) once a week for 4 weeks, the Ti contents were detected in the brain regions. Moreover, oxidative stress was induced, which led to inflammation and changed levels of neurotransmitters. Ultimately, mitochondria-mediated apoptosis were found.

Although low-dose exposure could not induce any acute neurotoxicity, chronic exposure to low dose TiO_2_ NPs might lead to dramatic damage to the brain. In Ze et al.’s study [85], the CD-1 female mice were treated with TiO_2_ NPs (5–6 nm; 2.5, 5, 10 mg/kg BW) every day for 90 days. The histopathological changes, including rarefaction of glial cells, dispersive replication of pyramidal cells, and reduced size of cell volume, were presented in the hippocampus. The neuronal ultrastructure was found to be affected as well, such as mitochondrial swelling and nuclear membrane collapse. TiO_2_ NPs-treated mice learned the training task more slowly than the control group and showed apparently downregulation of LTP amplitudes of fEPSP, which were consistent with the reduction in mRNA and protein levels of NR2A, NR2B, CREB-1, CREB-2, FosB/DFosB, and CaMKIV. These results suggested that the spatial recognition memory in mice should be impaired, due to long-term exposure of low-dose TiO_2_ NPs. Spatial recognition memory could be influenced by disturbance of the trace elements in mice’ s CNS due to chronic exposure to TiO_2_ NPs as well. In this study [86], CD-1 female mice were exposed to TiO_2_ NPs (5 nm; 5, 10, 50 mg/kg BW) every day for 60 days by intragastric administration. The Ti concentrations in the brain were upregulated in a dose-dependent way. The concentrations of Ca^2+^ and Na^+^ were markedly elevated, accompanied by decreased levels of Mg^2+^, K^+^, Zn^2+^, and Fe^3+^, all of which were consistent with reduction in the activities of Na^+^/K^+^-ATPase, Ca^2+^-ATPase, and C3a^2+^/Mg^2+^-ATPase of the brain. The levels of Ach, Glu, and NO were elevated, while the contents of NE, DA, DOPAC, 5-HT, and 5-HIAA were reduced. The disturbance in such elements, neurotransmitters, and enzymes may potentially impair the spatial recognition memory of experimental mice. In another research [87], male Wistar rats were intraperitoneally administrated with TiO_2_ NPs (20 mg/kg body weight) every 2 days for 20 days. This subacute exposure changed neurobehavioral performance of rats.

As mentioned above, TiO_2_ NPs are able to be translocated through the placenta barrier, influencing the fetal development. The fetal brain is another target of TiO_2_ NPs. Shimizu et al. [70] adopted DNA microarrays to determine altered genes expressions in the brain of male mice fetuses (ED 2) and pups (PD 2, 7, 14, 21) after their mothers were injected subcutaneously with TiO_2_ NPs (2570 nm; 100 μg, injection on gestational day (GD) 6, 9, 12, 15). They used GO category and MeSH term to analyze the microarray dataset and discovered that those changed genes were related with cell death, apoptosis, oxidative stress, inflammation, and neurotransmitters in the brain. This suggested that maternal exposure to TiO_2_ NPs would affect the brain development of fetuses and pups of mice. Cui et al. [72] found out that when pregnant Sprague–Dawley rats were exposed to TiO_2_ NPs (5 nm; 500 μl (1 μg/μl)) on gestational day (GD 6, 9, 12, 15, 18), the antioxidant capabilities of the brain in their pups would be affected. The levels of CAT, GSH-PX, and T-AOC in newborn pups were downregulated, while the level of MDA was enhanced in the experimental groups. The 8-hydroxydeoxyguanosine (8-OHdG) content was also upregulated, which might indicate that the maternal exposure to TiO_2_ NPs could induce oxidative stress to the nucleic acids in the fetal brain. Moreover, behavioral tests were finished during postnatal day (PD) 40 to 44. The experimental groups spent less time exploring the novel object and consumed less sucrose water. The time of immobility in the force swimming test was increased. These results inferred that the impairments in the brain of the offspring due to prenatal exposure to TiO_2_ NPs could affect the antioxidant capabilities of the newborn pups’ brain, leading to depressive-like behaviors during adulthood. In this study [66], when pregnant Wistar rats were treated with TiO_2_ NPs (10 nm; 100 mg/kg BW) every day from GD 2 to 21, the Ti contents were significantly elevated and the number of Ki-67-positive cells were reduced significantly in the hippocampus of PD 1 newborn as compared with the control group. Moreover, the learning and memory in offspring (PD21, PD60) were markedly disrupted. Yamashita et al. [67] had also demonstrated that after pregnant BALB/c mice were exposed to TiO_2_ NPs (35 nm, 0.8 mg) by intravenous injection on GD 16 and 17, Ti contents were detected in the placenta and fetal brain. Their uterine weights and fetuses sizes were reduced as well, leading to retarded development of the fetal brain.

#### Toxic effects on CNS in in vitro studies

In addition to in vivo studies, several in vitro researches (Table 3) investigated the neurotoxicity of TiO_2_ NPs as well. Xue et al. [88] treated primary microglia derived from Sprague–Dawley rats with 0.25 or 0.5 mg/ml TiO_2_ NPs (20 nm) for 24 or 48 h. The NO production was remarkably elevated, accompanied by increased mRNA and protein levels of iNOS. And the mRNA expressions of MCP-1 and MIP-1α were significantly enhanced in the experimental group as well. NF-κB binding activity was also increased markedly. However, the mRNA expression level of Th was significantly inhibited by TiO_2_ NPs. When PC12 cells were co-incubated with supernatant of TiO_2_ NPs-treated microglia for 48 h, the viability of PC12 cells, measured by MTT assay, was markedly reduced. In another study [89], PC12 cells were co-incubated with different concentrations of TiO_2_ NPs (range from 20 to 50 nm; 1, 10, 50, 100 μg/ml) for different times (6, 12, 24, 48 h). After 6 h incubation, only the 100 μg/ml TiO_2_ NPs-treated group showed significant reduction in cell viability. However, the viability of PC12 cells was decreased in all experimental groups except the 1 μg/ml after 12-, 24-, and 48-h incubation. Moreover, as the concentrations of TiO_2_ NPs increased, the percentage of dihydrodichlorofluorescein (DCF)-positive cells was elevated. Both the 10 and 50 μg/ml TiO_2_ NPs-treated groups demonstrated that there was an increased ratio of cell apoptosis after 24 h incubation. But pre-treated with N-MPG (ROS scavenger) could ameliorate the harmful effects on PC12 cells induced by TiO_2_ NPs. Wu et al. [90] also adopted PC12 cells as an model of dopaminergic neurons to study the neurotoxicity caused by TiO_2_ NPs on CNS. The PC12 cells were co-incubated with different concentrations of anatase or rutile TiO_2_ NPs (20 nm; 25, 50, 100, 200 μg/ml) for 24 h. The two crystal types induced apparent cytotoxicity. However, anatase TiO_2_ NPs led to significant lactate dehydrogenase (LDH) leakage at all concentrations. It induced significant elevation in DCF fluorescence intensity in 200 μg/ml anatase group, which was higher than rutile group with the same concentration. The levels of GSH and SOD were decreased by anatase (50, 100, 200 μg/ml) and rutile (200 μg/ml) TiO_2_ NPs, while the level of MDA was elevated by anatase (100, 200 μg/ml) and rutile (200 μg/ml) TiO_2_ NPs. Furthermore, the reduction in mitochondrial membrane potential in anatase (200 μg/ml) type was higher than that in rutile (200 μg/ml) one. Cell apoptosis and necrosis were also presented in TiO_2_ NPs-treated groups at the concentration of 200 μg/ml. The elevated percentage of G2/M phase cells was caused by anatase (100, 200 μg/ml) and rutile (200 μg/ml) TiO_2_ NPs as well. The protein levels of p-JNK, JNK, p-c-Jun, Jun, p-P53, p53, p21 GADD45, Bcl-2, and Bax were disrupted, too. The activity of caspase-3 was increased by anatase (50, 100, 200 μg/ml) and rutile (200 μg/ml) TiO_2_ NPs. Those changes might contribute to apoptosis or necrosis and cell cycle arrest in PC12 cells. In this study [91], primary hippocampal neurons from 1-day-old Sprague–Dawley rat were incubated with TiO_2_ NPs (approximate 5 nm; 5, 15, 30, 40, 50 μg/ml) for 6, 12, 24, or 48 h. The cytotoxicity, determined by MTT assay, demonstrated that the cell viabilities were reduced in a time-dependent and dose-dependent way. The LDH activity was significantly enhanced as well. Observed by TEM, it is revealed that changes of the ultrastructure of cells related with apoptosis were presented in cytoplasm and the apoptotic rate was elevated assessed by TUNEL method. Mitochondrial membrane potential (MMP) was markedly reduced, suggesting mitochondrial impairments. The Ca^2+^ concentration in cytoplasm was also apparently increased. Apoptotic cytokine levels, including cytochrome c, caspase-3, Bax, caspase-12, GRP78, and CHOP, were markedly enhanced with significant reduction in Bcl-2 level.

**Table 3:** Toxic effects of TiO2 NPs on CNS in in vitro studies

Crystal type	Cell type	Parameters/dose	Main findings	Reference
Unknown	Primary microglia from Sprague–Dawley rats and PC12	20 nm; 0.25 or 0.5 mg/ml; 24 or 48 h	NO, iNOS, MCP-1, MIP-1α, and NF-κB binding activity increased and Th inhibited in microglia; marked cytotoxicity in PC12 after incubation with supernatant of NPs-treated microglia	[88]
Anatase	PC12	Average 21 nm (range from 20 to 50 nm); 1, 10, 50, 100 μg/ml; incubated for 6, 12, 24, 48 h	Viability of cells decreased except 1 μg/ml group; DCF-positive cells and ratio of PC12 apoptosis elevated	[89]
Anatase and rutile	PC12	20 nm; 25, 50, 100, 200 μg/ml for 24 h	Apparent cytotoxicity; GSH, SOD, and mitochondrial membrane potential decreased; MDA and G2/M phase cells elevated; p-JNK, JNK, p-c-Jun, Jun, p-P53, p53, p21 GADD45, Bcl-2, and Bax disrupted	[90]
Anatase (96 %)	C6 U373	40–200 nm; 2.5, 5, 10, 20, 40 μg/ml; 24, 48 or 96 h	Apoptosis; cellular proliferation depressed; morphology and cytoskeleton changed; reduction in immune-location of F-actin fibers	[96]
Unknown	C6 U373	50 nm; 20 μg/cm^2^ for 2, 4, 6, 24, 48, 72 h	Imbalance in GPx, SOD and catalase; fluorescence of cis-parinaric acid and Rh123 downregulated; H2DCFDA and MitoTracker Green FM staining elevated	[97]
Rutile coated by SiO_2_	Mouse NSCs line C17.2	80–100 nm; 50, 100, 150, 200, 250 μg/ml exposed for 12, 24, 36, 48, 60, 72 h, or 7 days	Inhibition on cellular proliferation; β-tubulin positive cells detected; Cx43 elevated; PKCε reduced	[99]
Unknown	HCECs (human cerebral endothelial cells)	21 nm; 2 mg/ml; 0.12, 0.6, 3, 15, 75 μg/cm^2^ for 4, 24, 48, or 72 h	Significant cytotoxicity, ROS production, and marked DNA damage detected; cathepsin D and LC3-II upregulated	[98]
Anatase	Primary hippocampal neurons	5 nm; 5, 15, 30, 40, 50 μg/ml for 6, 12, 24, or 48 h	Cell viabilities and MMP reduced; LDH activities, apoptotic rate, and cytoplasmic Ca^2+^ elevated; ultrastructure of cells altered; apoptotic cytokine disturbed	[91]
Anatase (S) Anatase (80 %) + rutile (D)	Human SHSY5Y neuronal cells	25 nm; 20, 40, 60, 80, 100, 120, 140, 160 μg/ml for 3, 6, 24 h	No cytotoxicity; cell cycle changed; apoptotic cells elevated; genotoxicity detected; no oxidative damage	[92]
Anatase	Human neural stem cell line	80 nm; 0.01, 0.1, 1 mg/ml for 7 days	Morphology changed; mitochondrial activity not changed; Nestin, neurofilament heavy polypeptide, and high mobility group AT-hook 1 elevated	[100]

The function of human neuronal cells could be affected by TiO_2_ NPs. Valdiglesias et al. [92] treated human SH-SY5Y neuronal cells with anatase TiO_2_ NPs (TiO_2_-S) and TiO_2_ NPs (anatase (80 %) + rutile) (TiO_2_ NPs-D) at different concentrations (20, 40, 60, 80, 100, 120, 140, 160 μg/ml) for 3, 6, and 24 h. Cytotoxicity, assessed by MTT and NRU assays, was not induced by both types of TiO_2_ NPs, but NPs were apparently internalized by cells, observed by flow cytometry. Cell cycle, determined by analyzing the relative DNA content, was changed in the TiO_2_-S group. The elevated percentage of apoptotic cells, measured by flow cytometry, and genotoxicity, determined by Comet assay, were presented in both experimental groups. Both TiO_2_-S and TiO_2_-D induced no oxidative stress in human SH-SY5Y neuronal cells. Mao et al. [93] also employed the SH-SY5Y to investigate the neurotoxicity of TiO_2_ NPs. After the SH-SY5Y were exposed to TiO_2_ NPs (0.1, 1, 10, and 100 μg/ml), the cell viability was not affected in all groups. However, the microtubules of cells were disrupted, which contributed to the neurotoxic effects of TiO_2_ NPs. But a conflicting conclusion was obtained in a recent study [94]. After SH-SY5Y cell lines were treated with TiO_2_ NPs, the mitochondrial function was affected and cell membrane was damaged after both acute and chronic exposures. Hong et al. [95] reported that after the primary cultured hippocampal neurons were exposed to 5, 15, and 30 mg/ml nano-TiO_2_ for 24 h, the protein expressions of NMDAR were reduced. At the same time, the nitric oxide, nitrice synthase, and ADP/ATP ratios were upregulated. Those changes disrupted neurite outgrowth of hippocampal neurons.

As mentioned above, the glial cell could be another target of TiO_2_ NPs. In this study [96], C6 (rat’s glial cell) and U373 (human glial cell) were incubated with TiO_2_ NPs (40–200 nm; 2.5, 5, 10, 20, 40 μg/cm^2^) for 24, 48, or 96 h. The proliferation of C6 and U373 was depressed in a dose-dependent way after both cell lines were incubated with TiO_2_ NPs for 48 h. Morphological alterations of both cell lines were induced by TiO_2_ NPs after 96 h exposure at the concentration of 20 μg/cm^2^. TiO_2_ NPs were internalized by both cell lines after 24 h incubation which was observed by TEM. The immune-locations of F-actin fibers in C6 and U373 were observed after 24 and 96 h exposure. Those data demonstrated that the fluorescence of F-actin in C6 and U373 was decreased and the degrees of this reduction were closely related with concentration, exposure time, and cell type. Moreover, apoptosis was induced by TiO_2_ NPs in both cell lines, which was determined by DAPI nuclear staining. In another study [97], C6 and U373 were incubated with TiO_2_ NPs (50 nm; 20 μg/cm^2^) for 2, 4, 6, 24, 48, or 72 h. Data obtained from the research demonstrated that the H2DCFDA oxidation in both cell lines was significantly enhanced by treatment with TiO_2_ NPs for 2 h, reaching its maximum at 6 h, and was reduced at 24 h. The mRNA levels of antioxidant enzyme, including GPx, catalase, and SOD, were determined by RT-PCR, which demonstrated that their expressions were elevated at an early exposure time and then decreased at a later period in both C6 and U373. Reduction in the fluorescence of cis-parinaric acid and Rh123 indicated oxidation of lipids and disturbance of mitochondrial function, respectively. The mitochondrial depolarization was assessed by MitoTracker Green FM, and the staining was markedly increased in both cell lines after treatment with TiO_2_ NPs for 24 and 48 h. These results suggested that TiO_2_ NPs were able to cause oxidative stress and the mitochondrion could be impaired in both C6 and U373 glial cell lines in vitro study.

Once NPs were absorbed into the circulation system after exposure, they have to cross the BBB to enter into the brain regions. Therefore, the integrity of the BBB could be affected by NPs as well. In this in vitro study [98], human cerebral endothelial cells (HCECs) were exposed to 21-nm TiO_2_ NPs at different concentrations for different times. After 24 h incubation, TiO_2_ NPs were internalized by HCECs which were observed by TEM. Significant cytotoxicity reduction was determined by MTT assay after 72 h exposure. Carboxy-H2DCFDA demonstrated that TiO_2_ NPs induced apparent elevation in ROS production after 4 h treatment. Marked DNA damage was also observed in cells by Comet assay after 24 and 48 h incubation. Moreover, the levels of activated cathepsin D and LC3-II were upregulated, which indicated that TiO_2_ NPs induced autophagy.

The TiO_2_ NPs still have some positive effects on the brain in addition to neurotoxicity. In this research [99], mouse neural stem cells (NSCs) line C17.2 were incubated with TiO_2_ NPs (coated with SiO_2_; 80–100 nm; 50, 100, 150, 200, 250 μg/ml) for 12, 24, 36, 48, 60, 72 h, or 7 days to determine the effects of TiO_2_ NPs on the differentiation trend of neural stem cells. It was discovered that after C17.2 cells were incubated with TiO_2_ NPs for 7 days, the β-tubulin positive cells were obviously increased as compared with that in the control group. This finding indicated that the TiO_2_ NPs could induce the C17.2 differentiating into neurons. But once the HB1.F3 human neural stem cells (hNSCs) were incubated with TiO_2_ NPs (80 nm; 0.01, 0.1, 1 mg/ml) for 7 days, the cells were aggregated and the morphology of cells changed with no change in mitochondrial activity. The levels of proteins, linked to hNSC differentiation including Nestin (stem cell marker) and neurofilament heavy polypeptide (N-FH; neuron marker), were elevated with the increase in mRNA level of high mobility group AT-hook 1 (HMGA1) after 24 h exposure [100].

We can draw a conclusion from the abovementioned in vivo and in vitro researches that for the typical properties of TiO_2_ NPs, exposure to them might pose a high risk on the brain health. Molecular mechanisms underlying the neurotoxicity of TiO_2_ NPs might mainly include oxidative stress, apoptosis, inflammation, and disturbance of ATPases or neurotransmitters. Similarly, these sorts of mechanisms could be present in other types of nanomaterials besides TiO_2_ NPs [101–104]. But what factors mainly influence the neurotoxicity of TiO_2_ NPs are still unclear.

### Major factors influence the neurotoxicity of TiO_2_ NPs

The neurotoxic effects of TiO_2_ NPs could be modulated by its peculiar physicochemical characteristics, administration routes, dosage, and so on. Therefore, although the abovementioned studies were all focused on the harmful impacts of TiO_2_ NPs on the brain, different conclusions had been obtained. As a consequence, for the purpose of assessing their neurotoxicity in a standard way, it is vital to discuss the major factors that might influence the neurotoxicity of TiO_2_ NPs.

#### Crystal type

TiO_2_ NPs, unlike other NPs, have two crystal types, i.e., the anatase and rutile [31]. Both of them possess subtle different physicochemical characteristics, which lead to different toxicities. It was reported that the toxicity of anatase form was higher than that of rutile [105–107]. However, some studies did not draw the same conclusions [108, 109]. Concerning the TiO_2_ NPs, how different crystal forms of TiO_2_ NPs affect neurotoxicity is still unclear. In this study [25], CD-1 (ICR) female mice were treated with anatase (155 nm) or rutile (80 nm) TiO_2_ NPs. The levels of GFAP protein, MDA, AChE activity, and glutamic acid in anatase group were higher than that in rutile group. However, the numbers of cell lost in both groups were similar, with no statistical difference. In another report of the same research group [53], the elevated levels of GSH-Px, GST, GSH, and SOD in rutile group were significantly higher than that in control and anatase groups at the time point of 10 days after CD-1 (ICR) female mice were treated with TiO_2_ NPs. But the levels of IL-1β and TNF-α in anatase group were apparently higher than that in the control and rutile groups after 30 days exposure. The rutile TiO_2_ NPs did not induced elevation in the levels of IL-1β and TNF-α.

PC12 cells were exposed to anatase or rutile TiO_2_ NPs (20 nm), the reduction of cell viability, mitochondria membrane potential (MMP), and levels of GSH and SOD in the anatase group were remarkably higher than that in the rutile one at the concentration of 200 μg/ml. The levels of LDH and MDA, the caspase 3 activity, and the percentage of necrosis in the anatase group were increased, which were higher than that in the rutile group at the concentration of 200 μg/ml [90]. However, an inconsistent conclusion was drawn from this study that the effects of both TiO_2_-S (100 % anatase NPs) and TiO_2_-D (80 % anatase + 20 % rutile) on the CNS were similar with no statistical difference [92].

#### Size of NPs

Several researches have revealed that dimension of nanomaterial is another vital factor which can influence the nanotoxicity [110]. When it comes to the effects of TiO_2_ NPs on the CNS, little is known about how different sizes affect the neurotoxicity of TiO_2_ NPs. Available data collected from current researches only compared the toxicity of the micro-TiO_2_ with that of nanosized-TiO_2_. Micro-TiO_2_ was found to be not detected in the brain regions. Thus, it was demonstrated to have no toxic effects [59, 79, 82, 90, 100]. Therefore, for fully understanding the neurotoxicity of TiO_2_ NPs, more studies are needed to investigate the effects of TiO_2_ NPs on the CNS with different sizes at the nanoscale level.

#### Administration route

As for in vivo studies, the administration routes play an important part on neurotoxicity of NPs [111, 112]. The Ti contents in the brain regions of mice/rats could not be detected when the TiO_2_ NPs were administrated via intravenous injection [73–75, 113]. But when the pregnant mice were treated with TiO_2_ NPs, their uterine weights were lowered with smaller fetuses, which indirectly suggested that the TiO_2_ NPs could induce fetal resorption and retard fetal growth via intravenous injection [67]. Ti could not be detected in the brain when mice were exposed to TiO_2_ NPs by inhalation [114]. Based on current studies, the intranasal instillation or nasal administration was the most effective pathway for TiO_2_ NPs to be translocated into the brain [16]. This might be due to the retrograde axonal translocation of NPs directly from the nose to the brain. Therefore, more researches are needed to investigate how and why the different administration routes lead to different NP bio-distributions.

#### Shape and Surface modification

The morphology of NPs is crucial to their toxicity. The effects of NPs on organisms might be regulated by morphological structure [115–119]. However, how different shapes (mainly including nanobelts, nanorods, nanotubes, and nanospheres) of TiO_2_ NPs modulate their transportation to the brain, how they get excretion from the CNS, and how they have toxic effects on neurons or glia cells are still largely unknown. Moreover, the surface coating can regulate the physicochemical properties of TiO_2_ NPs, which might influence their toxicity [120, 121]. However, whether TiO_2_ NPs coated with inorganic or organic materials could alleviate or exacerbate the harmful impacts on the CNS is unclear as well. In this study [99], mouse NSCs line C17.2 were treated with SiO_2_-coated rutile TiO_2_ NPs. This exposure induced elevation of the β-tubulin positive cells, which indicated that the TiO_2_ NPs can induce the C17.2 differentiating into neurons. In another study [100], the HB1.F3 human neural stem cells (hNSCs) were incubated with uncoated TiO_2_ NPs for 7 days, the cells were aggregated and the morphology of cells was changed. Results from the two researches might suggest that the coating on TiO_2_ NPs could make NPs possess beneficial properties. Zhang et al. [59] investigated how different sizes, morphology, and surface modification of TiO_2_ NPs in rutile form regulated their toxic effects on the brain after CD-1 (ICR) female mice were exposed by intranasal instillation every other day for 30 days. The experimental groups included A (micro-sized, hydrophobic, rod-like, and no coating), B (nano-sized, hydrophobic, needle-like, and no coating), C (nano-sized, hydrophilic, needle-like, and coated with silica), and D (nano-sized, hydrophilic, rod-like, and coated with silica). Results collected from the study demonstrated that (1) micro-sized TiO_2_ could not be detected in the brain regions. The Ti contents in the cerebellum region demonstrated no significant difference in the five groups (four experimental groups and one control group). Significantly increased Ti contents in the cerebral cortex were detected in C and D groups. In the striatum region, the Ti contents were markedly elevated in the B, C, and D groups; (2) the neuron loss in cerebral cortex and hippocampus CA1 region was significant in B, C, and D groups; (3) the levels of norepinephrine in the hippocampus, cerebral cortex, cerebellum, and striatum were significantly reduced in groups C and D; and (4) the levels of DA, DOPAC, HVA, 5-HT, and 5-HIAA in the four sub-brain regions were significantly affected in groups C and D. These findings suggested that the size, shape, and surface modification could modulate the toxic effects of TiO_2_ NPs in the brain. However, studies about effects of shape and surface modification on neurotoxicity of TiO_2_ NPs were limited, which needed further investigations.

## Conclusions

As the rapid development of nanotechnology, numerous nanomaterial-based products are widely used at present, such as consumer products, food additives, cosmetics, drug carriers, and so on. Meanwhile, concerns on health risks about unexpected exposure to TiO_2_ NPs are arising. In in vivo studies, once animals were exposed to TiO_2_ NPs, the NPs could be translocated into the brain mainly through the blood–brain barrier (BBB) and nose–brain pathway. Besides, TiO_2_ NPs may affect the brain development of embryo by crossing the placental barrier. The Ti contents accumulated in the brain regions are tiny at one exposure, but its elimination from the brain was limited. Therefore, long-term or chronic exposure to TiO_2_ NPs could potentially lead to the gradually increased Ti contents in the brain, which may eventually induce impairments on the neurons and glial cells and lead to CNS dysfunction as a consequence.

Several in vivo and in vitro studies have demonstrated that TiO_2_ NPs, for their nanoscale, possessed toxic properties on the brain. However, as the experimental parameters used in all of the current studies were not standardized (such as different administration routes, experimental animals, crystal forms, different shapes and sizes), the conclusions from those studies are not comparable and even some of them might be conflicting. Therefore, it is urgent to standardize experiments on assessing the neurotoxicity of TiO_2_ NPs. In addition, all the research objects in those experiments were only consisted of animals (such as mice and rats) or cells (such as PC12, U373, C6). In consequence, neurotoxic data of TiO_2_ NPs collected from those studies might be inappropriate to determine their neurotoxic effects on humans. Therefore, in order to fully understand the neurotoxicity of TiO_2_ NPs, using human exposures or cells derived from humans to do experiment are needed. On the other hand, the toxic effects of different physicochemical characteristics of TiO_2_ NPs on the brain are unclear and should be investigated intensively, which includes the crystal forms, shape, size, surface modifications, and so on. In order to reduce translocation rate of TiO_2_ NPs into the brain and neurotoxicity, it is urgent to seek out the optimum parameters of physicochemical properties of TiO_2_ NPs to improve the bio-safety of TiO_2_ NPs-based products. Moreover, because TiO_2_ NPs could induce neurons or glial cells death and disturb the homeostasis in the brain, the possible relationship between the TiO_2_ NPs exposure and neurodegenerative diseases or psychiatric disorders needs further investigation.
